# *HK1*–*LDHA* axis feedback regulation promotes histone lactylation of *Wnt5a* and mediates prenatal acetaminophen-induced susceptibility to osteoarthritis in male offspring

**DOI:** 10.1038/s12276-026-01793-1

**Published:** 2026-07-27

**Authors:** Fan Zhang, Qingxian Li, Xiaoxiang Sun, Yu Guo, Hui Wang, Liaobin Chen

**Affiliations:** 1 Department of Orthopedic Surgery, Division of Joint Surgery and Sports Medicine, Zhongnan Hospital of Wuhan University, Wuhan, China; 2 Department of Pharmacology, Wuhan University School of Basic Medical Sciences, Wuhan, China; 3 Hubei Provincial Key Laboratory of Developmentally Originated Disease, Wuhan, China; 4 Present Address: Department of Orthopedic Surgery, First Affiliated Hospital of Xiamen University, School of Medicine, Xiamen University, Xiamen, China

**Keywords:** Genetics research, Cartilage development, Dosage compensation, Predictive markers

## Abstract

Acetaminophen is widely used during pregnancy but may cause developmental abnormalities in multiple systems in offspring. However, the effects of prenatal acetaminophen exposure (PAcE) on chondrodevelopment and long-term outcomes remain unclear. We administered 100 mg/kg per day of acetaminophen to rats on gestational days (GDs) 10–12 and treated fetal chondrocytes in vitro. In male PAcE offspring, cartilage matrix synthesis decreased and degradation increased at GD20 and postnatal week 12, with osteoarthritis (OA) susceptibility after running. Females exhibited only reduced matrix content at GD20. Mechanistically, acetaminophen inhibited the expression of hexokinase 1 (*HK1*) in chondrocytes under both normoxic and hypoxic conditions in vitro. However, *HK1* inhibition induced feedback enhancement of the glycolytic only under hypoxic conditions, which increased lactic acid production and H3K18la lactylation levels in the *Wnt5a* promoter region, disrupting cartilage homeostasis. These effects were reversed by *Wnt5a* knockdown, *HK1* overexpression, or *LDHA* knockdown. Finally, intra-articular adeno-associated virus-sh*Wnt5a* injection improved OA pathology in male PAcE offspring. In summary, PAcE upregulated H3K18la levels with *Wnt5a* through *HK1-LDHA*-mediated glycolytic feedback regulation, causing chondrodysplasia and increased OA susceptibility in male offspring, with sex differences. Our findings provide crucial insight into the hypoxia-dependent chondrotoxicity of PAcE, providing a reference for future research on fetal-derived OA.

**Figure:**
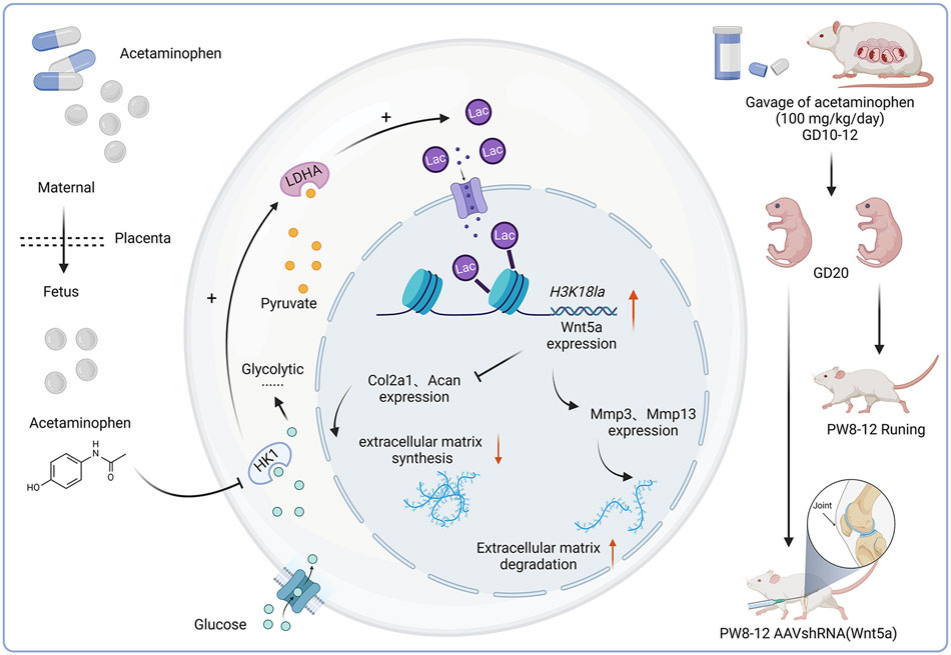
High H3K18la in the *Wnt5a* promoter region mediates PAcE-induced hypochondroplasia and susceptibility to adult OA in male offspring rats.

High H3K18la in the *Wnt5a* promoter region mediates PAcE-induced hypochondroplasia and susceptibility to adult OA in male offspring rats.

## Prenatal acetaminophen alters offspring cartilage development

Osteoarthritis (OA) is a degenerative joint disease affecting millions globally, with emerging evidence linking its origin to prenatal environments. This study explores how prenatal acetaminophen exposure (PAcE) affects cartilage development and OA susceptibility in offspring. Using a rat model, researchers found that PAcE disrupts fetal cartilage matrix synthesis and increases degradation, particularly in male offspring, leading to adult OA susceptibility. The study identifies Wnt5a, a key protein in cartilage development, as a mediator of these effects, with its expression upregulated by PAcE. The researchers used adeno-associated virus to knock down Wnt5a, which restored cartilage balance, highlighting its potential as a therapeutic target. The study also reveals that PAcE alters glycolysis in chondrocytes, increasing lactate levels and histone lactylation, which epigenetically upregulates Wnt5a. These findings suggest that careful acetaminophen use during pregnancy could mitigate OA risk. This summary was initially drafted using artificial intelligence, then revised, and fact-checked by the author.

## Introduction

Osteoarthritis (OA) is a degenerative joint disease characterized by articular cartilage deterioration and contributes substantially to the global burden of pain and disability. Its pathogenesis involves multifactorial mechanisms, including mechanical, inflammatory, metabolic, and genetic factors^1^. Globally, OA affected approximately 595 million people (7.6% of the population) in 2020, representing a 1.5-fold increase in total cases since 1990 (refs. ^2–4^). Notably, OA onset increasingly affects younger populations, with cases manifesting as early as adulthood^5^. Epidemiological evidence and genetic analyses suggest that the origin of OA in adults may lie in the intrauterine environment^6,7^. In recent years, with the proposal and in-depth exploration of the “Developmental origins of health and disease (DOHaD)” theory, the relationship between the prenatal environment and fetal organ development has received increasing attention^8^. Animal studies confirm that adverse gestational exposures induce chondrodysplasia^9–11^, supported by our previous findings that prenatal xenobiotic exposure causes offspring chondrodysplasia and predisposes to adult OA under stressors^12–14^. In conclusion, OA has intrauterine developmental origins.

Acetaminophen (paracetamol), the most commonly prescribed analgesic during pregnancy for fever and mild-to-moderate pain^15^, is considered the lowest risk and the highest benefit analgesic during gestation^16^. However, acetaminophen’s transplacental passage and persistence in the fetal circulation exacerbate developmental toxicity^17^. Recent research indicates that gestational analgesic use (acetaminophen, ibuprofen, and aspirin) increases the risks of adverse outcomes, including low birth weight, neurodevelopmental delays, and reproductive system abnormalities^18–21^. Our recent study demonstrated that high-dose prenatal acetaminophen exposure (PAcE) administered in mid-gestation reduces fetal murine cartilage matrix content, disrupts metabolic homeostasis, and induces chondrodysplasia^22^. Collectively, these findings indicate that acetaminophen poses multisystem developmental toxicity risks and increases susceptibility to adult diseases and is closely associated with skeletal system dysplasia. However, whether and how PAcE affects intrauterine cartilage development and postnatal susceptibility to OA remains unclear.

Epigenetic regulation governs embryonic cartilage formation and adult OA pathogenesis^23,24^. Cellular metabolism directly supplies the substrates for these epigenetic modifications. For instance, acetyl-CoA, a key energy metabolite, serves as a substrate for histone acetylation and modulates the transcription of cartilage development-related genes^23^. Our previous studies detected elevated lactic acid, a glycolytic metabolite, in fetal blood following gestational xenobiotic exposure (for example, nicotine and dexamethasone)^25,26^. Lactate is not merely a metabolic by-product; it acts as a direct substrate for histone lactylation to activate gene transcription^27^. Abnormal lactate accumulation and histone lactylation are closely associated with musculoskeletal disorders and disease progression^28–32^. However, whether PAcE induces local lactic acid accumulation in fetal cartilage and histone lactylation-mediated gene dysregulation, thereby impacting developmental chondrogenesis and adult OA susceptibility, remains unclear.

In this study, we established a rat model of fetal-derived OA induced by PAcE combined with postnatal long-distance running to identify therapeutic targets for PAcE-induced offspring articular chondrodysplasia and adult OA susceptibility. We elucidated PAcE-induced histone hyper lactylation in fetal cartilage genes and clarified its underlying mechanism. These findings support rational use of acetaminophen during pregnancy and provide novel etiological insights and preventive strategies for fetal-derived OA.

## Materials and methods

### Chemicals and reagents

Acetaminophen (No. MB1698) and Opti-MEM (PWL041) were purchased from Meilun Biotechnology Co., Ltd (Dalian, China). Isoflurane was obtained from Baxter Healthcare Co., Ltd (Deerfield, IL, USA). TRIzol reagent (SKU: R6830-02) was acquired from Omega Bio-Tek Co., Ltd (Doraville, GA, USA). Reverse transcription and real-time quantitative PCR (RT-qPCR) kits and SYBR Green dye were obtained from Vazyme Biotechnology Co., Ltd (Nanjing, China). All of the primers for the gene used for analysis were synthesized by TIANYIHUIYUAN Biotechnology Co., Ltd (Wuhan, China). Safranin O (G1053-1), fast green (G1053-2), anti-collagen type II alpha 1 chain (COL2A1) (GB11021), anti-aggrecan (ACAN) (GB11373), anti-matrix metallopeptidase 3 (MMP3) (GB11131), anti-HIF-1α (GB151339), 4′,6-diamidino-2-phenylindole (DAPI) (G1012), tris-buffered saline with tween (TBST) (G0004), and DAB Color Development Kit (G1212) were obtained from Servicebio Co., Ltd (Wuhan, China). Anti-hexokinase 1 (HK1) (A23524), anti-H3 (A17562), anti-β-actin (AC004), horseradish peroxidase (HRP) goat anti-rabbit IgG (H+L) (AS014), and fluorescein Isothiocyanate goat anti-rabbit IgG (H+L) (AS011) were purchased from ABclonal Technology Co., Ltd (Wuhan, China). Anti-wingless-type mmtv integration site family member 5a (Wnt5a) (55184-1-AP) and anti-matrix metallopeptidase 13 (MMP13) (18165-1-AP) were purchased from ProteinTech Biotechnology Co., Ltd (Wuhan, China). PTM Biotechnology Co., Ltd (Hangzhou, China) provided the anti-Pan-Kla (PTM-1401RM), anti-H3K9la (PTM-1419RM), anti-H3K14la (PTM-1414RM), and anti-H3K18la (PTM-1406RM). Lactic acid detection kit was purchased from Nanjing Jiancheng Bioengineering Research Institute Co., Ltd (Nanjing, China). DMEM/F12, phosphate-buffered saline (PBS), trypsin-EDTA, and fetal bovine serum (A5670701) were purchased from Gibco (Grand Island, NY, USA). Other chemicals and agents were of analytical grade.

### Animals and treatments

All of the animal experiments were performed in the Center for Animal Experiments of Wuhan University, which is accredited by the Association for the Assessment and Accreditation of Laboratory Animal Care International. All of the animal experimental procedures were approved by the Committee on the Ethics of Animal Experiments of the Wuhan University School of Medicine (Permit No. WP20240035).

Specific pathogen-free Wistar rats (male: 280 ± 20 g and female: 220 ± 20 g) were obtained from the Experimental Center of the Hubei Medical Scientific Academy (certificate no.: 2024025004630, license no. SCXK2020-0018). Rats were fed under standard environment and allowed to have normal diet. All the rats were fed adaptively for a week before the experiment. A male rat and two female rats were caged together overnight for mating. The day when the sperm are observed on the vaginal image, it is regarded as the gestational day (GD) 0. Pregnant rats were randomly allocated into groups as follows: ①CON: 10 ml/kg vehicle administered intragastrically (i.g.) on GD10–12 and ②PAcE: 100 mg/kg per day acetaminophen administered i.g. on GD10–12. The drug was administered at 8 p.m. once a day. There were 12 pregnant rats in each group. Some pregnant rats were given birth naturally, and the offspring were subjected to long-distance running modeling at the postnatal 8th week (PW8). Then knee joints were obtained at PW12.

Pregnant rats were anesthetized with 2% isoflurane on GD20, and the hind limbs of fetuses were obtained immediately. Three articular samples of fetal rats from different litters were fixed within 4% paraformaldehyde for pathological examination, and the remaining samples were immediately frozen at −80 °C for gene analysis. Three articular cartilages from each litter were randomly chosen as one sample, and 8–12 samples from different litters were included in each group for gene analysis. In addition, adult offspring were sacrificed on PW12 and the knees were fixed within 4% paraformaldehyde, and the remaining samples were immediately frozen at −80 °C for subsequent gene analysis. At the same time, genes related to the shape and function of articular cartilage were detected, and the quality of articular cartilage and OA was evaluated. Finally, 8-week-old offspring rats induced by PAcE were included in the in vivo intervention experiments.

### Adeno-associated virus (AAV) injection in knee joint

AAV vector serotype 9 (AAV9) (with a *COL2A1* promoter) is the preferred viral vector to target chondrocytes after injection. AAV9 *Wnt5a* short hairpin RNA (shRNA) sequence was cloned into the pAV-*COL2A1*-GFP-mir30-shRNA to get *Wnt5a*-RNAi plasmid. Specific sequences and nonspecific controls were separately constructed in one vector, and AAV9 harboring these sequences was generated by Vigene Biosciences (Jinan, China). Rats were injected with one of the above viruses for 4 weeks before knee joint specimens were obtained and subsequent experimental testing was carried out. Rats were divided into four groups (*n* = 6): Control+AAV-sh negative control (NC), Control+AAV-sh*Wnt5a*, PAcE+AAV-shNC, and PAcE+AAV-sh*Wnt5a* groups. The AAV injected into the knee joint of rats was provided by Vigene Biosciences. The sequence of the shRNA is AGGCGTGGCTATGACCAGTTTATAGTGAAGCCACAGATGTATAAACTGGTCATAGCCACGCCC.

### In vivo bioluminescence imaging

Animals were placed in a small animal vital imager (Bruker, Germany) after anesthesia with isoflurane and then imaged using a small animal in vivo imaging system (Xtreme B1, Bruker). Imaging was performed in fluorescence mode under the following parameters: modality, fluorescence; session type, current; light source, multiwavelength; reference file, none. Excitation and emission wavelengths were selected according to the fluorescent plasmid label used: mScarlet (excitation 569 nm and emission 594 nm) or GdGreen (excitation 469 nm and emission 506 nm). Additional settings included an exposure time of 2 s, a field of view of 10 cm, an f-stop of 2, and a focal plane set at 1.1 mm. All other parameters were maintained at default values. Image data were analyzed using Bruker MI SE software (Bruker).

### Safranin O/fast green staining

For histological analysis, the knee joints were fixed in 4% paraformaldehyde and embedded in paraffin. The specimens were sectioned sagittally at a thickness of 6 μm. The slices were stained with fast green for 5 min and wash off the excess dye with water, then stained with safranin O for 10–20 s. The specimens were dehydrated quickly with anhydrous ethanol after safranin O staining. At last, the images were photographed with an Olympus AH-2 light microscope (Olympus, Tokyo, Japan). ImageJ software was used to calculate the integrated optical density (IOD) of images in an area of 300 × 300. We evaluated the IOD with two different fields for each sample. Sample preservation follows the aforementioned experimental steps.

### Immunohistochemistry (IHC) analysis

Knee joints were fixed in 4% paraformaldehyde for 24 h and then embedded in paraffin. Serial longitudinal sections at 6 μm were prepared. IHC assays were performed to determine the protein expression of COL2A1, ACAN, MMP3, MMP13, and Wnt5a in the articular cartilage. Briefly, after deparaffinized in xylene, antigen retrieval with boiling in sodium citrate buffer, and incubated in 3% H_2_O_2_ for 20 min, sections were blocked in 3% bovine serum albumin for 30 min followed by incubation with the primary antibody (1:200 for COL2A1, ACAN, MMP3, MMP13, and Wnt5a) in a humidified chamber at 4 °C overnight. A biotinylated secondary antibody was added for 1 h on the second day and then followed by an avidin-biotinylated HRP complex according to the manufacturer’s protocol. Finally, peroxidase activity was revealed by immersion in DAB substrate. The histological images were captured using an Olympus AH-2 light microscope (Olympus). ImageJ software was used to calculate the IOD in two different fields for each sample.

### Immunofluorescence (IF) analysis

IF assay was performed to determine the protein expression of Wnt5a and Pan-Kla in the articular cartilage. Knee joints were fixed in 4% paraformaldehyde for 24 h and then embedded in paraffin. Serial longitudinal sections at 6 μm were cut. After antigen retrieval with boiling in sodium citrate buffer, sections were then blocked in 3% bovine serum albumin for 30 min. Primary antibody of anti-Wnt5a (1:200) and anti-Pan-Kla (1:200) was added and incubated in a humidified chamber at 4 °C overnight. Fluorescent secondary antibody (1:100) was added for 1 h at 25 °C and then incubated with DAPI for 15 min at 25 °C in darkness. Images were captured using a Nikon NIS Elements BR light microscope (Nikon, Tokyo, Japan). ImageJ software was used to calculate the mean optical density in two different fields for each sample.

Meanwhile, similar method was used to detect the expression of Wnt5a and HIF-1α proteins in primary chondrocytes. The details are as follows: primary chondrocytes were uniformly planted in a 20 mm confocal dish. When the cell density reached 50–60%, acetaminophen was treated for 72 h. The chondrocytes were gently washed three times with precooled PBS. Then 1 ml of 4% paraformaldehyde fixing solution was added to each dish. After 15 min, the fixing solution was discarded and washed three times with PBS. The chondrocytes were permeated with 0.2% Triton 100 for 10 min, then blocked for 1 h, and finally incubated with the primary antibody solution of anti-Wnt5a (1:200) and anti-HIF-1α (1:200) overnight in the dark. All subsequent steps were carried out under dark conditions. On the next day, the cells were washed three times with TBST for 10 min each time and incubated with secondary antibody solution for 1 h. After the cells were washed three times with TBST, DAPI solution was added. After incubation in dark for 10 min, they were washed with PBS three times for 5 min each time. Finally, IF images of the cells were taken using a laser confocal microscope (Leica, Germany).

### Fetal cartilage RNA-sequencing (RNA-seq)

According to the protocol of manufacturers, the total RNA was isolated from fetal cartilage at GD20 using TRIzol Reagent (Thermo Fisher Scientific Inc., USA). RNA integrity was assessed using the RNA Nanodrop (Thermo Fisher Scientific Inc.). Then, mRNA was purified from total RNA using poly-T oligo-attached magnetic beads. Two-stranded cDNA was synthesized after mRNA fragmentation, using random hexamer primer and M-MuLV Reverse Transcriptase (RNase H) and DNA Polymerase I and RNase H, respectively. To select cDNA fragments of preferentially 370–420 bp long, the library fragments were purified with AMPure XP system (Beckman Coulter, Beverly, MA, USA). Then PCR was performed with Phusion High-Fidelity DNA polymerase, Universal PCR primers. PCR products were purified (AMPure XP system) and library quality was assessed on the Agilent Bioanalyzer 2100 system. Finally, the clustering of the index-coded samples was performed on a cBot Cluster Generation System using TruSeq PE Cluster Kit v3-cBot-HS (Illumina), according to the instructions of manufacturers. After cluster generation, the library preparations were sequenced on an Illumina Novaseq platform and 150 bp paired-end reads were generated (Novgene Biotech Co., Ltd, Beijing, China).

### Total RNA extract and RT-qPCR

Total RNA of fetal articular cartilage was isolated by TRIzol reagent, following the manufacturer’s protocol. cDNA was amplified using a one-step RT-qPCR reaction. Relative mRNA expression levels were normalized against *β-ACTIN*. The rat primer sequences are shown in Table 1.

**Table 1:** Oligonucleotide primer sequences for real-time quantitative PCR.

Genes	Forward primer sequences (5’ → 3’)	Reverse primer sequences (5’ → 3’)
*β-ACTIN*	GTGACGTTGACATCCGTAAAGA	GCCGGACTCATCGTACTCC
*COL2A1*	GCAGAATGGGCAGAGGTATAA	ACCAGCCTTCTCGTCAAATC
*ACAN*	GGACAGTAGTGCGGACATTAG	CAGTAGGGAAACCTGAGACAAG
*MMP3*	CTGTGTGCTCATCCTACCC	CAGGGCTACTGTCCTTTTT
*MMP13*	GTGCCTGATGTGGGTGAATA	GGCCCATCAAATGGGTAGAA
*WNT5A*	GATGCCCTCAAGGAGAAGTATG	CGGTCTGCACCGTCTTAAA
*HK1*	TGGGCTTCACCTTCTCATTTC	CTCCACCATCTCCACATTCTTC
*PFKM*	ATCACAGCCGAGGAGGCTAC	GGCGGCCCATCACTTCTAAC
*PKM*	ATGGCTGACACATTCCTGGAGC	CCTTCAACGTCTCCACTGATCG
*PEPCK*	CTGCATAATGGTCTGGACTTCT	GTCTCTCTGCTCTTGGGTAATG
*LDHA*	CAAACTCCAAGCTGGTCATTATC	GAGGGTATCTGCACTCTTCTTC

*ACAN* aggrecan, *COL2A1* collagen type II alpha 1 chain, *HK1* hexokinase 1, *LDHA* lactate dehydrogenase A, *MMP3* matrix metallopeptidase 3, *MMP13* matrix metallopeptidase 13, *PEPCK* phosphoenolpyruvate carboxykinase, *PFKM* phosphofructokinase, *PKM* pyruvate kinase.

### Cell treatment and culture

The cells used in in vitro experiments in this study were all primary chondrocytes, which were extracted from rat knee cartilage within 1 week of birth. The specific steps mainly include peeling off the skin and muscles of the rat knee joint layer by layer, then separating the knee joint cartilage, chopping the obtained knee joint cartilage, and placing it in 2% type 2 collagenase for 6–8 h, and mixing and shaking every 2 h to ensure adequate digestion of the tissue. Then, the digested medium was centrifuged to obtain primary chondrocyte precipitates, which were then transferred to DMEM/F12 medium containing 10% fetal bovine serum for cell expansion. All vitro experiments were performed after two generations. When the cells reached 80% confluence, different concentrations of acetaminophen dissolved in dimethyl sulfoxide (DMSO) were added to 6-well plates (final concentration: 0.1% DMSO), and the same volume of control medium containing 0.1% DMSO was added to the control cells. In this study, cell culture was carried out in two oxygen environments: hypoxia and normoxia. The incubator in the normoxia group was maintained at 21% O_2_ and 5% CO_2_, whereas the incubator in the hypoxia group was maintained at 1% O_2_ and 5% CO_2_. For each independent experiment, at least three technical replicates were used. To evaluate the effect of exogenous lactate supplementation, cells were treated with lactate for 48 h and 72 h under hypoxic conditions. Lactate was added directly to the culture medium at the time of medium change. Control group received an equal volume of vehicle (culture medium without lactate). After the treatment, cells were harvested for subsequent assays.

### Cellular silencing and overexpression of genes

The small interfering RNA (siRNA) targeting the junction region of lactate dehydrogenase A (*LDHA*), and *Wnt5a* sequence and *HK1* overexpression plasmid were designed and synthesized by Jima Biotechnology Co., Ltd (Suzhou, China). *HK1* overexpression, *LDHA*, and *Wnt5a* silencing was achieved by transfecting the primary chondrocytes using Lipofectamine 3000 and P3000 (Thermo Fisher Scientific Inc.), and transfection reagent was provided by Jima Biotechnology Co., Ltd. Primary rat chondrocytes were cultured in 6-well plates in complete DMEM/F12 medium and grown to 70–80% confluence in a monolayer. The transfection of siRNA or plasmid was performed with Lipofectamine 3000 and P3000. An aliquot of 5 μl of siRNA (2 μg plasmid + 5 μl P3000) + 125 μl of Opti-MEM media and 5 μl of Lipofectamine 3000 + 125 μl of Opti-MEM medium was mixed separately in two Eppendorf tubes. After incubation for 5 min, the samples of above two tubes were mixed gently and incubated for 20 min at room temperature. After 6–8 h of culture, the medium was changed to normal complete medium to continue cultivating the cells and performing subsequent molecular biology experiments. The sequence of the siRNA of *LDHA* is 5ʹ-3ʹ GCCAUCAGUAUCUUAAUGATT and 3ʹ-5ʹ UCAUUAAGAUACUGAUGGCTT and that of siRNA of *Wnt5a* is 5ʹ-3ʹ GGUCCCUAGGUAUGAAUAATT and 3ʹ-5ʹ UUAUUCAUACCUAGGGACCTT.

### Cell viability analysis

As described earlier, primary chondrocytes extracted from rat knee joint cartilage were uniformly planted in a 96-well plate, with a cell density of approximately 5000 cells/100 ml per well. After cells were adhered to the wall, different concentrations of acetaminophen (0, 10, 20, and 40 μM) were given for 48 h and 72 h. After the incubation, the medium was discarded and replaced with 10% cell counting kit-8 (CCK-8) (MA0218) (Meilun Biotechnology Co., Ltd) solution. After incubation in an incubator at 37 °C for 2 h, it was placed in a spectrophotometer for detection. The absorbance at 450 nm wavelength was measured with a microplate reader (Bio-Rad, Hercules, CA, USA).

### Seahorse glycolytic proton efflux rate assay

Glycolytic proton efflux rate (glycoPER) was measured using a Seahorse XFe96 analyzer with the Glycolytic Rate Assay Kit (Agilent). Primary chondrocytes were seeded at 2 × 10^4^ cells/well in XF96 plates and cultured under hypoxic conditions. On the assay day, cells were washed and incubated in a non‑CO_2_ incubator for 45 min. Rotenone/antimycin A (Rot/AA) (0.5 μM) was injected to block mitochondrial respiration, followed by 2‑deoxy‑D‑glucose (2-DG) (50 mM) to inhibit glycolysis. Basal glycoPER was derived from the pre-injection period, and compensatory glycoPER was determined after Rot/AA injection (before 2‑DG). All values were normalized to cell number. Three independent experiments were performed with six replicates per condition.

### Western blotting (WB) analysis

WB was used to measure the expression of protein in the research. The general steps are as follows: after cells and cartilage tissues are collected, radioimmunoprecipitation assay (RIPA) (P0013B) (Beyotime Biotechnology Co., Ltd, Shanghai, China) lysis solution containing 1% phenylmethanesulfonyl fluoride (PMSF) is added, after ultrasonically or milling, left on ice for 20 min, and centrifuged at 10,000×*g* and 4 °C for 10 min. Pipette the supernatant and add 1/4th of the solution volume Loading buffer (Biosharp Biotechnology Co., Ltd, Beijing, China), shake and mix well, heat at 100 °C for 10 min to denature the protein, and finally obtain a protein solution for subsequent experiments. After the protein solution was separated by SDS–PAGE, the membrane was transferred at a constant current of 300 mA to transfer the protein to the polyvinylidene fluoride (PVDF) membranes (05317-10EA) (Merck Millipore, Germany), according to the specific molecular weight. The PVDF membranes were then blocked in a rapid blocking solution for 20 min, and then the blocked PVDF membranes were placed in the diluted primary antibody solution (H3 (1:10,000), H3K9la (1:1,000), H3K14la (1:1,000), H3K18la (1:1,000), Pan Kla (1:1,000), and Wnt5a (1:1,000)) and incubated at 4 °C overnight. The next day, the PVDF membranes were washed three times with TBST for 10 min each time. After washing, it was mixed with HRP-labeled secondary antibody (goat anti-rabbit antibody 1:10,000 and goat anti-mouse antibody 1:10,000). Incubated for 1 h at room temperature, TBST is used to wash the PVDF membranes three times for 10 min each and finally developed with a highly sensitive enhanced chemiluminescence kit (34579) (Thermo Fisher Scientific Inc.). WB imaging was obtained with Tanon 5200 automatic chemiluminescent image analyzer (Tianneng, Shanghai, China). Images were acquired and quantitative analysis was performed using ImageJ software, and each experiment was repeated three times.

### Chromatin immunoprecipitation (ChIP) analysis

In this study, chromatin immunoprecipitation technology was used to detect the binding of proteins to the DNA promoter region. The specific steps are as follows: first, the cells were collected after trypsinizing, and 1% formaldehyde was added to crosslink the chromatin. The tissues were added with 1% formaldehyde and crushed in a mill and incubated for 15 min. Secondly, glycine was added to terminate the crosslink, incubated at room temperature for 10 min, centrifuged at 3,000×*g* at 4 °C for 5 min, discarded the supernatant, and then placed the beads, antibodies, and chromatin on a rotating shaker to incubate at 4 °C overnight, while taking a certain amount of solution as input. On the second day, the immunoprecipitate was washed and the chromatin–DNA complex was uncrosslinked and decrosslinked in a 65 °C-water bath overnight together with the input solution. On the third day, the immunoprecipitate was followed by DNA purification kit (Beyotime Biotechnology Co., Ltd). The instructions purified the samples and finally carried out RT-qPCR experiments on the promoter region of the target gene. UCSC Genome Browser was used to design the DNA promoter region amplification primers of the target gene Wnt5a. Input and IgG were used as internal control and NC, respectively, to conduct data analysis. Finally, the protein binding situation in the promoter region of the target gene is obtained.

### Liquid chromatography–mass spectrometry (LC–MS) analysis

Targeted quantification of methanol-resuspended polar metabolites was conducted using a HPLC system interfaced with a TSQ Quantis triple quadrupole mass spectrometer (Thermo Fisher Scientific Inc.). Analyte separation used a Hypersil GOLD C18 column (Thermo Fisher Scientific Inc.). The mobile phase consisting of 0.1% (v/v) methanol in water (for other metabolites) delivered at a constant flow rate of 0.200 ml/min. Acquired data were processed utilizing Xcalibur software (Thermo Fisher Scientific Inc.).

### Molecular docking

Molecular docking was performed to assess the binding interactions between acetaminophen and the candidate target proteins. Before docking, all pre-existing ligands and water molecules were removed from the protein structures using PyMOL (DeLano Scientific LLC, USA). The docking simulations were carried out with Vina software (Scripps Research Institute, USA). On the basis of the scoring function implemented in Vina, lower binding energy scores indicate stronger binding affinity between the ligand and the receptor.

### Statistical analysis

SPSS 20 (SPSS Science Inc., Chicago, IL, USA) and Prism 9 (Graph Pad Software, La Jolla, CA, USA) were used for data statistics and graphing. Experimental data were expressed as the mean ± SEM. Student’s unpaired two-tailed *t* test and one-way analysis of variance were used to compare the mean values of two or various groups as applicable. Statistical significance was noted as follows: *P* < 0.05 versus corresponding control.

## Results

### PAcE induces OA susceptibility in adult offspring rats with fetal developmental origin and sex-specific differences

**Fig. 1:**
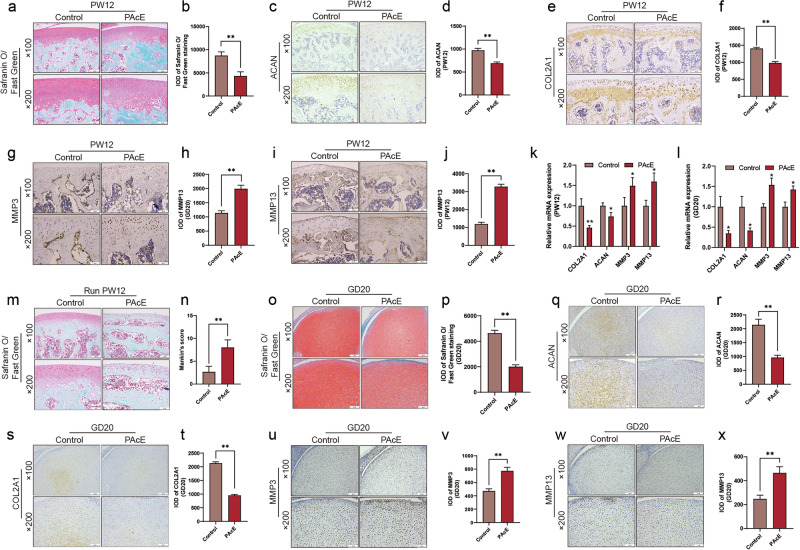
PAcE induces dyschondroplasia and OA susceptibility in adult male rats. **a**, **b** Representative images of safranin O/fast green staining of PW12 cartilage and quantification of IOD values; scale bars, 100 μm and 50 μm, *n* = 5 biological replicates. **c**–**j**, Representative images of immunohistochemical staining for COL2A1, ACAN, MMP3, and MMP13 in fetal cartilage and quantification of IOD values; scale bars, 100 μm and 50 μm, *n* = 5 biological replicates. **k**, **l** The mRNA expression of *COL2A1*, *ACAN*, *MMP3*, and *MMP13* at GD20 and PW12, *n* = 8–12 biological replicates. **m**, **n** Representative images of safranin O/fast green staining and quantification of their IOD values of PW12 and long-distance running cartilages treated with model creation; scale bars, 100 μm and 50 μm, *n* = 5 biological replicates. **o**–**x** Representative pictures of immunohistochemical staining and statistics of their IOD values of fetal cartilages COL2A1, ACAN, MMP3, and MMP13 at GD20; scale bars, 100 μm and 50 μm, *n* = 5 biological replicates. All experiments were performed in technical triplicate. Values are expressed as mean ± SEM. ^*^P < 0.05, ^**^P < 0.01 versus corresponding control. *ACAN*, aggrecan; *COL2A1*, collagen type II alpha 1 chain; GD20, gestational day 20; IOD, integrated optical density; *MMP3*, matrix metallopeptidase 3; *MMP13*, matrix metallopeptidase 13; OA, osteoarthritis; PAcE, prenatal acetaminophen exposure; PW12, postnatal week.

On the basis of clinically relevant dosages and previous animal experiments, we found that acetaminophen exposure at 400 mg/kg per day during the second trimester of pregnancy induced chondrodysplasia in fetal mice^22^. Furthermore, following the “two-hit” theory, PAcE offspring were subjected to long-distance running during PW8–12. Compared with the control group, safranin O/fast green staining intensity and IOD value in the PAcE group were significantly reduced (Fig. 1a, b). The mRNA and protein expression of matrix synthesis-related genes *COL2A1* and *ACAN* was markedly decreased, whereas the mRNA and protein expression of degradation-related genes *MMP3* and *MMP13* was increased (Fig. 1c–k). Moreover, compared with the control group subjected to long-distance running, the PAcE group exhibited more pronounced cartilage damage, characterized by reduced cartilage thickness, disorganized chondrocyte arrangement, and increased Mankin’s score (Fig. 1m, n). The effect of PAcE on the morphological and functional development of fetal cartilage in male offspring (GD20) was further examined. Safranin O/fast green staining showed that cartilage in the PAcE group was irregular and lighter in color, and the IOD value was significantly lower than that of the control group (Fig. 1o, p). In addition, the mRNA and protein expression of *COL2A1* and *ACAN* in the PAcE group was significantly decreased, whereas the expression of *MMP3* and *MMP13* was increased (Fig. 1l, q–x). In conclusion, PAcE reduces cartilage matrix synthesis and enhances matrix degradation in male offspring rats both prenatally and postnatally, leading to chondrodysplasia and increased OA susceptibility in adulthood.

**Fig. 2:**
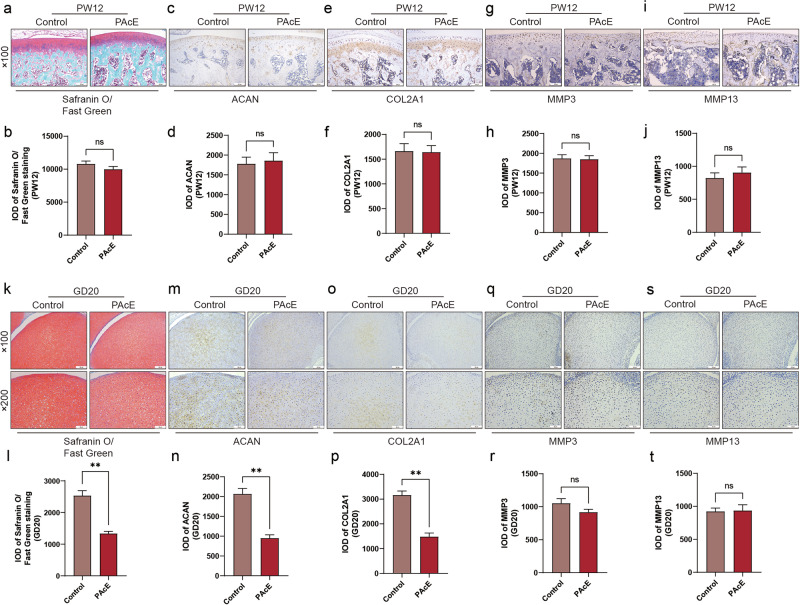
Effect of PAcE on prenatal and postnatal cartilage development in female offspring rats. **a**, **b** Representative images of safranin O/fast green staining of PW12 cartilage and its IOD value statistics; scale bar, 100 μm, *n* = 5 biological replicates. **c**–**f** Representative images of immunohistochemical (IHC) staining of PW12 cartilage COL2A1 and ACAN and quantification of IOD value; scale bar, 100 μm, *n* = 5 biological replicates. **g**–**j** Representative images of IHC staining of PW12 cartilage MMP3 and MMP13 and quantification of IOD value; scale bar, 100 μm, *n* = 4 biological replicates. **k**, **l** Representative images of safranin O/fast green staining of fetal cartilage at GD20 and quantification of IOD value. **m**–**p** Representative images of IHC staining of fetal cartilage COL2A1 and ACAN at GD20 and their IOD value statistics; scale bars, 100 μm and 50 μm, *n* = 5 biological replicates. **q**–**t** Representative images of IHC staining of fetal cartilage *MMP3* and *MMP13* and quantification of IOD value; scale bars, 100 μm and 50 μm, *n* = 5 biological replicates. All experiments were performed in technical triplicate. Values are expressed as the means ± SEM. ^*^P < 0.05, ^**^P < 0.01 versus corresponding control. *ACAN*, aggrecan; *COL2A1*, collagen type II alpha 1 chain; GD20, gestational day 20; IOD, integrated optical density; *MMP3*, matrix metallopeptidase 3; *MMP13*, matrix metallopeptidase 13; ns, not significant; OA, osteoarthritis; PAcE, prenatal acetaminophen exposure; PW12, postnatal week.

Meanwhile, we examined the phenotypic and functional changes in cartilage in female offspring rats. At PW12, compared with the control group, there were no significant changes in cartilage safranin O/fast green staining, protein expression of COL2A1 and ACAN, as well as MMP3 and MMP13 in the PAcE group (Fig. 2a–j). At GD20, the safranin O/fast green staining in the PAcE group appeared lighter, and the IOD value decreased (Fig. 2k, l), the protein expression of COL2A1 and ACAN was also reduced (Fig. 2m–p), whereas MMP3 and MMP13 expression did not change significantly (Fig. 2q–t). These findings indicates that PAcE reduces the cartilage matrix synthesis in female offspring rats before birth without affecting the degradation pathway and does not impair cartilage structure or function at PW12. In conclusion, PAcE induces OA susceptibility in offspring rats with a fetal developmental origin in a sex-specific manner, predominantly affecting male offspring.

### *Wnt5a* mediates cartilage changes in prenatal and postnatal male offspring rats induced by PAcE

**Fig. 3:**
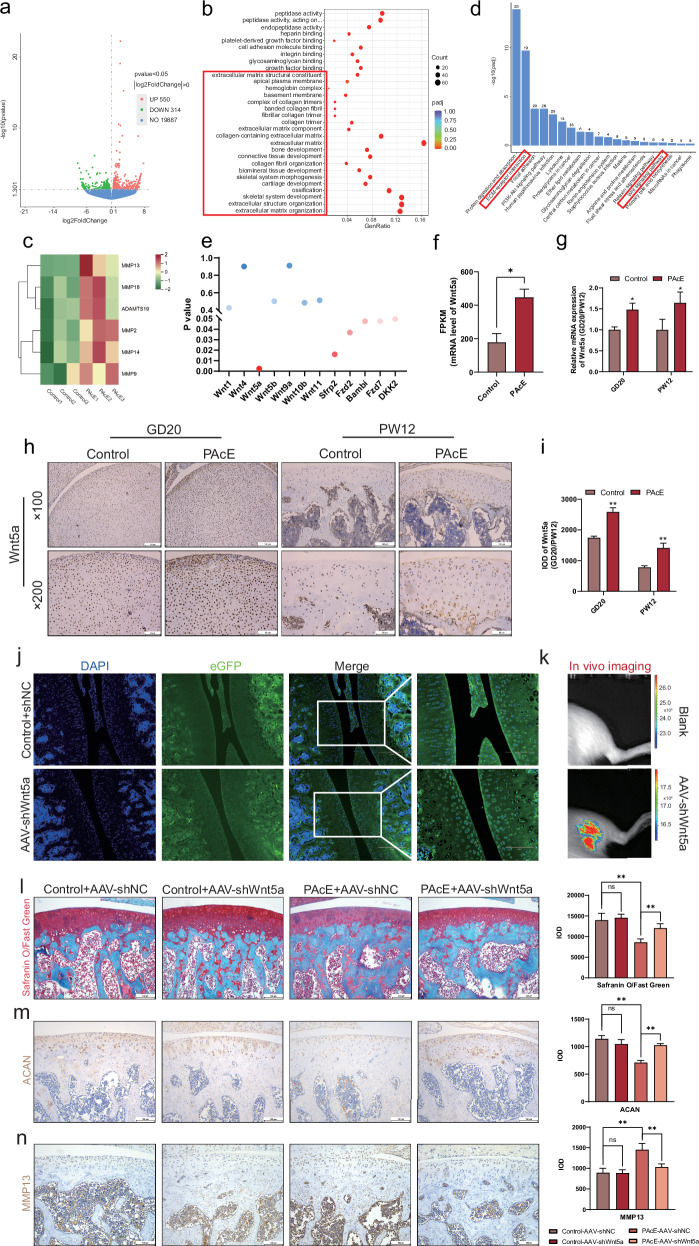
*Wnt5a* mediates prenatal and postnatal cartilage alterations in male offspring rats following PAcE exposure. **a**, **b** Volcano plot and GO enrichment analysis of differentially expressed genes from fetal cartilage RNA-seq data. **c** Foldchange of matrix degradation-related genes (*MMP9* and *MMP13*) in fetal cartilage RNA-seq data. **d** KEGG pathway enrichment analysis of differentially expressed genes. **e** *P*-values of Wnt pathway ligands from RNA-seq data. **f** Foldchange of *Wnt5a* expression in fetal cartilage RNA-seq data. **g**–**i** *Wnt5a* mRNA levels (*n* = 8–12 biological replicates) and representative immunohistochemical staining with IOD quantification in GD20 and PW12 cartilage; scale bars, 100 μm and 50 μm, *n* = 5 biological replicates. **j** Representative eGFP staining in knee joints of AAV-shNC and AAV-sh*Wnt5a* groups; scale bars, 300 μm and 150 μm, *n* = 5 biological replicates. **k** Representative in vivo imaging of knee joints from Blank and AAV-sh*Wnt5a* groups. **l** Representative safranin O-fast green staining of knee sections in Control+AAV-shNC, Control+AAV-sh*Wnt5a*, PAcE+AAV-shNC, and PAcE+AAV-sh*Wnt5a* groups; scale bar, 100 μm, *n* = 5 biological replicates. **m**, **n** Representative IHC staining of ACAN and MMP13 in knee sections across experimental groups; scale bar, 100 μm, *n* = 5 biological replicates. All experiments were performed in technical triplicate. Values are expressed as means ± SEM. ^*^P < 0.05, ^**^P < 0.01 versus corresponding control. AAV, adeno-associated virus; *ACAN*, aggrecan; *COL2A1*, collagen type II alpha 1 chain; DAPI, 4′,6-diamidino-2-phenylindole; GD20, gestational day 20; GO, Gene Ontology; IOD, integrated optical density; KEGG, Kyoto Encyclopedia of Genes and Genomes; *MMP3*, matrix metallopeptidase 3; *MMP13*, matrix metallopeptidase 13; MOD, mean optical density; ns, not significant; OA, osteoarthritis; PAcE, prenatal acetaminophen exposure; PW12, postnatal week; shNC, short hairpin negative control; sh*Wnt5a*, short hairpin wingless-type mmtv integration site family member 5a; *SMAD2*, SMAD family member 2; *SMAD4*, SMAD family member 4; *Wnt5a*, wingless-type mmtv integration site family member 5.

On the basis of the aforementioned findings, we further explored the intrauterine programming mechanism underlying PAcE-induced dyschondroplasia in male offspring rats and their susceptibility to adult OA. First, we performed transcriptome sequencing of GD20 fetal cartilage tissue. Among the differentially expressed genes, 550 were upregulated and 314 were downregulated (Fig. 3a). Gene Ontology (GO) analysis revealed significant enrichment of biological pathways related to extracellular matrix tissue, cartilage development, collagen fiber tissue, and collagen catabolism (Fig. 3b). The mRNA expression of extracellular matrix tissue-related genes such as SMAD family member 2 (*SMAD2*) and SMAD family member 4 (*SMAD4*) was significantly decreased, whereas the mRNA expression of collagen catabolism genes such as matrix metallopeptidase 9 (*MMP9*) and *MMP13* was significantly increased (Fig. 3c). These results suggest that PAcE decreases fetal cartilage matrix synthesis and increases matrix degradation in male offspring. Furthermore, Kyoto Encyclopedia of Genes and Genomes enrichment analysis identified upregulation of the Wnt signaling pathway, which is closely associated with cartilage matrix metabolism (Fig. 3d). Among the Wnt pathway ligands involved in cartilage differentiation, *Wnt5a* exhibited the most significant change in expression (Fig. 3e, f). We confirmed that the mRNA and protein expression of *Wnt5a* in PAcE offspring rats was significantly increased at GD20 and PW12 (Fig. 3g–i). To further verify whether *Wnt5a* acts as a toxic target, we used AAV targeting chondrocytes to perform a local *Wnt5a* gene knockdown operation on the knee cartilage of PAcE male offspring. IF and in vivo imaging confirmed successful transfection of knee joint cartilage by AAV (Fig. 3j, k). The IHC images showed that compared with the Control+AAV-shNC group, the protein expression of Wnt5a in the knee cartilage of the Control+sh*Wnt5a* group was significantly decreased (Supplementary Fig. 1a, b). Meanwhile, compared with the PAcE+AAV-shNC group, the protein expression of Wnt5a in the knee cartilage of the PAcE+AAV-sh*Wnt5a* group was significantly reduced (Supplementary Fig. 1a, b). This indicates that AAV successfully knocked down the protein expression of Wnt5a in the knee cartilage. Safranin O/fast green staining revealed that, compared with the Control+AAV-shNC group, cartilage matrix content in the PAcE+AAV-shNC group was significantly decreased (Fig. 3l), whereas, compared with the PAcE+AAV-shNC group, the cartilage matrix content in the PAcE+AAV-sh*Wnt5a* group was significantly increased (Fig. 3l). Furthermore, compared with the Control+AAV-shNC group, the protein expression of ACAN in the PAcE+AAV-shNC group decreased, whereas the protein expression of MMP13 significantly increased (Fig. 3m, n). Compared with the PAcE+AAV-shNC group, ACAN protein expression increased and MMP13 protein expression decreased in the PAcE+AAV-sh*Wnt5a* group (Fig. 3m, n). Together, these findings demonstrate that the sustained prenatal and postnatal overexpression of *Wnt5a* mediates chondrodysplasia and susceptibility to OA in male offspring and serves as a key toxic target contributing to reduce cartilage matrix synthesis and enhance matrix degradation.

### *Wnt5a* overexpression disrupts matrix metabolism in primary fetal chondrocytes under acetaminophen exposure

**Fig. 4:**
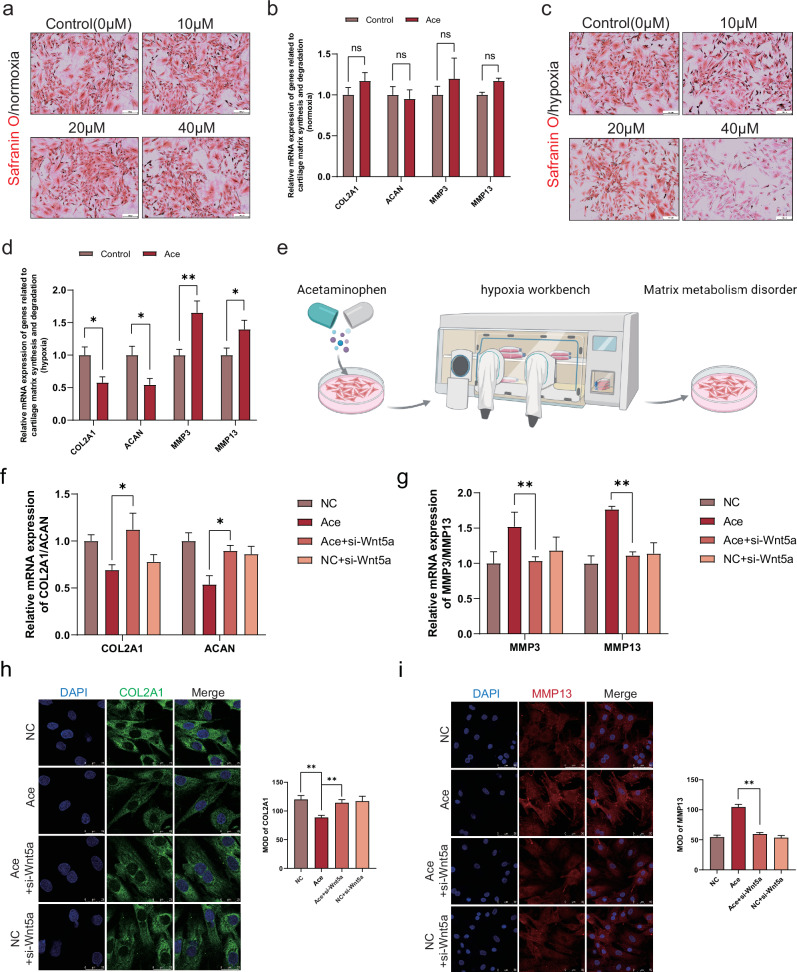
*Wnt5a* overexpression disrupts matrix metabolism in primary fetal chondrocytes exposed to acetaminophen under hypoxic conditions. **a** Safranin O staining images of chondrocytes treated with different acetaminophen concentrations; scale bar, 100 μm, *n* = 6 biological replicates. **b** mRNA expression of *COL2A1*, *ACAN*, *MMP3*, and *MMP13* in chondrocytes treated with different acetaminophen concentrations under normoxic conditions, *n* = 6 biological replicates. **c** Safranin O staining images of chondrocytes treated with different acetaminophen concentrations, scale bar, 100 μm, *n* = 6 biological replicates. **d** mRNA expression of *COL2A1*, *ACAN*, *MMP3*, and *MMP13* of chondrocytes treated with different acetaminophen concentrations under hypoxia conditions, *n* = 6 biological replicates. **e** Schematic diagram of cell culture under normoxic and hypoxic environments. **f**, **g** mRNA expression of *COL2A1*, *ACAN*, *MMP3*, and *MMP13* in primary fetal chondrocytes treated with acetaminophen and *Wnt5a*-siRNA, *n* = 6 biological replicates. **h**, **i** Representative IF images of COL2A1 and MMP13 in primary fetal chondrocytes treated with acetaminophen and *Wnt5a*-siRNA and their statistical analysis; scale bar, 25 μm, *n* = 6 biological replicates. All experiments were performed in technical triplicate. Values are expressed as means ± SEM. ^*^P < 0.05, ^**^P < 0.01 versus corresponding control. *ACAN*, aggrecan; Ace, acetaminophen; *COL2A1*, collagen type II alpha 1 chain; DAPI, 4′,6-diamidino-2-phenylindole; IF, immunofluorescence; LC–MS, liquid chromatography–mass spectrometry; *MMP3*, matrix metallopeptidase 3; *MMP13*, matrix metallopeptidase 13; MOD, mean optical density; NC, negative control; ns, not significant; *Wnt5a*, wingless-type mmtv integration site family member 5a; *Wnt5a*-siRNA, silencing-wingless-type MMTV integration site family member 5a.

We further investigated the mechanism underlying the sustained prenatal and postnatal changes in *Wnt5a* expression. The blood concentration of acetaminophen in PAcE fetal blood was measured by LC–MS. The results showed that acetaminophen levels in the fetal blood of PAcE male offspring ranged from 12.6 μM to 23.6 μM. On the basis of these results, we set the treatment concentrations for in vitro experiments to 0, 10, 20, and 40 μM. Under normoxia culture conditions, acetaminophen had no significant effect on cartilage matrix synthesis or degradation (Fig. 4a, b), which was inconsistent with the in vivo findings. Considering that chondrocytes in vivo reside in a hypoxic environment, we repeated the experiment under hypoxic conditions. Compared with the control group, acetaminophen reduced the mRNA expression of *COL2A1* and *ACAN* and upregulated the mRNA expression of *MMP3* and *MMP13* (Fig. 4c–e), consistent with the in vivo results. These findings suggest that acetaminophen disrupts chondrocyte matrix homeostasis under hypoxic conditions in vitro, and its toxic effect on chondrocytes depends on the hypoxic environment. Subsequently, we further explored whether the activation of *Wnt5a* mediated these matrix metabolic changes. The acetaminophen-induced decrease in *COL2A1* and *ACAN* expression and the upregulation of *MMP3* and *MMP13* were reversed by transfection with *Wnt5a*-siRNA (Fig. 4f, g). Similarly, cellular IF staining confirmed that *Wnt5a*-siRNA restored COL2A1 expression and reduced MMP13 expression compared with acetaminophen treatment alone (Fig. 4h, i). Together, these results indicate that, under hypoxia conditions, acetaminophen reduces matrix synthesis and enhances matrix degradation in primary fetal chondrocytes through upregulating *Wnt5a* expression.

### *HK1* mediates the enhancement of cartilage glycolysis in male offspring rats induced by PAcE

**Fig. 5:**
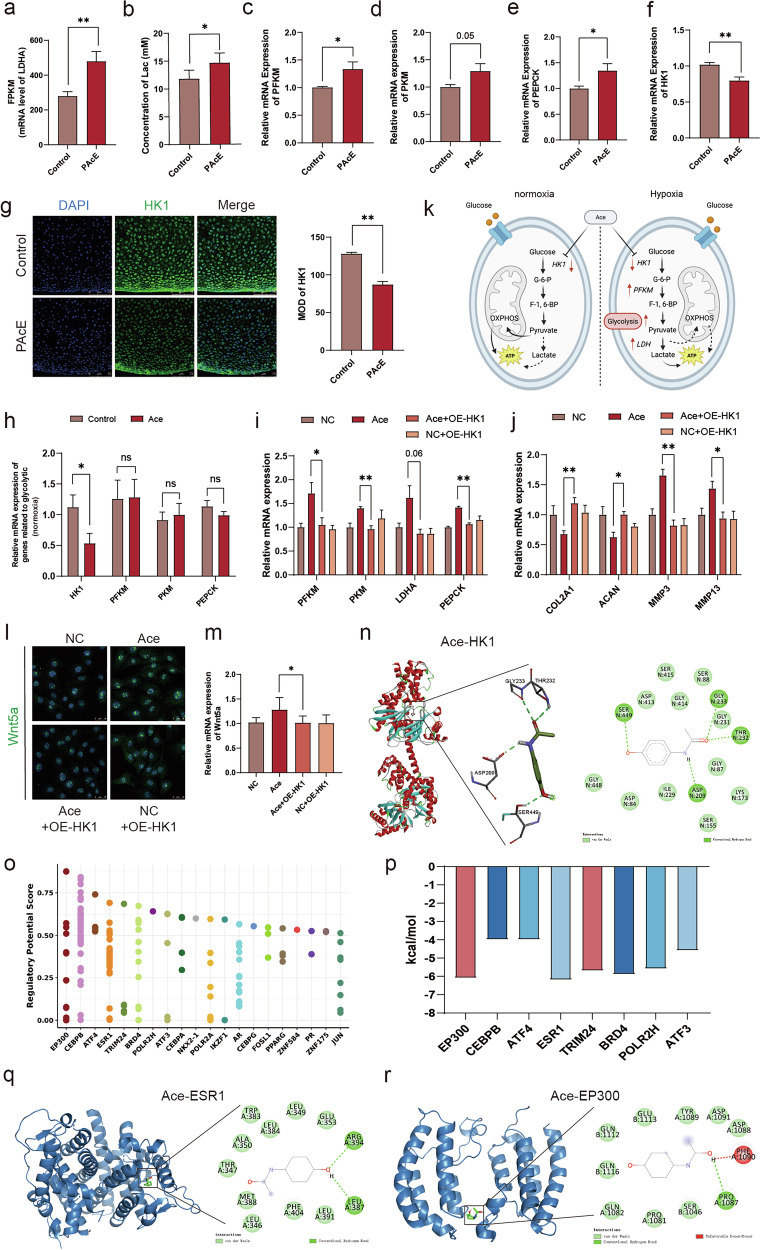
*HK1* mediates the enhancement of glycolysis in fetal chondrocytes induced by acetaminophen. **a** FPKM value of *LDHA* in RNA-seq. **b** Lactic acid content in fetal cartilage. **c**–**e** mRNA expression of glycolysis downstream genes in fetal cartilage, *n* = 8–12 biological replicates. **f** The mRNA expression of *HK1* in fetal cartilage, *n* = 8–12 biological replicates. **g** Representative IF images of HK1 in fetal cartilage; scale bar, 75 μm, *n* = 6 biological replicates. **h** mRNA expression of glycolysis downstream genes in fetal cartilage; *n* = 8–12 biological replicates. **i**, **j** Effect of *HK1* overexpression on the mRNA expression of *PFKM*, *PKM*, *LDHA*, *PEPCK*, *COL2A1*, *ACAN*, *MMP3*, and *MMP13* in primary fetal chondrocytes under hypoxic conditions, *n* = 6 biological replicates. **k** Flow chart of feedback enhancement of glycolysis of chondrocytes under hypoxia conditions. **l**, **m** Effect of overexpression of *HK1* on mRNA and protein expression of Wnt5a, *n* = 6 biological replicates. **n** Visual analysis of molecular docking between acetaminophen and HK1. **o** Transcription factors predicted in Cistrome that may regulate *HK1*. **p** Statistics on the binding energy of the molecule docking between acetaminophen and transcription factors predicted to possibly regulate HK1. **q**, **r** Visualization of the docking of *ESR1* and *EP300* with acetaminophen molecules, respectively. All experiments were performed in technical triplicate. Values are expressed as means ± SEM. ^*^P < 0.05, ^**^P < 0.01 versus corresponding control. ALA, alanine; ASP, aspartic acid; *ATF3*, activating transcription factor 3; *ATF4*, activating transcription factor 4; *BRD4*, bromodomain containing 4; CEBPB, CCAAT enhancer binding protein beta; DAPI, 4′,6-diamidino-2-phenylindole; EP300, E1A binding protein P300; *ESR1*, estrogen receptor 1; FPKM, fragments per kilobase million; GD20, gestational day 20; GLN, glutamine; GLU, glutamic acid; GLY, glycine; *HK1*, hexokinase 1; Lac, lactic acid; LEU, leucine; MET, methionine; MOD, mean optical density; NC, negative control; OXPHOS, oxidative phosphorylation; OE, overexpression; PAcE, prenatal acetaminophen exposure; *PEPCK*, phosphoenolpyruvate carboxykinase; *PFKM*, phosphofructokinase; PHE, phenylalanine; *PKM*, pyruvate kinase M1/2; POLR2H, RNA polymerase II, I, and III subunit H; PRO, proline; SER, serine; THR, threonine; THR, threonine; TRIM24, tripartite motif containing 24; TRP, tryptophan; TYR, tyrosine; Wnt5a, wingless-type mmtv integration site family member 5a.

The differential effects of acetaminophen on cartilage phenotypes under hypoxic and normoxic conditions described earlier suggest that the abnormal cartilage matrix metabolism caused by acetaminophen is closely related to the hypoxic environment. Cartilage in a hypoxic environment relies primarily on glycolysis to sustain cellular metabolism and biosynthesis^33^. On the basis of this, we analyzed the glycolysis-related pathways in the transcriptome sequencing data and found that the foldchange value of the key glycolytic gene *LDHA* in the PAcE group was significantly increased (Fig. 5a). Previous studies have shown that alterations in glycolysis and lactic acid production were closely associated with cartilage matrix metabolism and development^28,34,35^. Consistent with these findings, we found that compared with the control group, the lactic acid content in cartilage tissue of the PAcE group was increased at GD20 (Fig. 5b), and the mRNA expression of key downstream genes *PFKM*, *PKM*, and *PEPCK* were upregulated (Fig. 5c–e). However, both the mRNA and protein expression of the rate-limiting glycolytic enzyme gene *HK1* was significantly downregulated in the cartilage tissue of the PAcE group (Fig. 5f, g). Interestingly, under normoxic conditions, acetaminophen also reduced the mRNA expression of *HK1*, whereas the mRNA expression of *PFKM*, *PKM*, and *PEPCK* did not change significantly (Fig. 5h, i). Under hypoxic conditions, overexpression of *HK1* reversed the acetaminophen-induced upregulation of *PFKM*, *PKM*, *PEPCK*, and *LDHA* (Fig. 5j), as well as the changes in *COL2A1*, *ACAN*, *MMP3*, and *MMP13* expression (Fig. 5k). The results of the glycolysis rate detection showed that the overexpression of *HK1* alleviated the increase in glycoPER caused by Ace (Supplementary Fig. 2a). Furthermore, the IF results of HIF-1α under hypoxic conditions showed that, compared with the control group, there were no significant changes in the expression and nuclear translocation of *HIF-1α* in the Ace group (Supplementary Fig. 2b). The results above suggest that PAcE inhibits *HK1* expression under hypoxic conditions, which in turn enhances glycolysis and increases lactate content in chondrocytes through negative feedback regulation, and *HIF-1α* does not participate in the stress response induced by Ace under hypoxic conditions. However, the inhibitory effect of acetaminophen on *HK1* expression appeared independent of oxygen tension (Fig. 5k). Moreover, overexpression of *HK1* reversed the acetaminophen-induced increase in *Wnt5a* mRNA and protein expression (Fig. 5l, m), indicating that *HK1* inhibition mediated the upregulation of *Wnt5a* expression caused by acetaminophen.

To explore the potential mechanism by which acetaminophen affected *HK1* expression, we performed molecular docking simulations between acetaminophen and the HK1 protein using AutoDock software. The results showed that the binding energy of the acetaminophen-HK1 docking was −5.7 kcal/mol (less than −5 kcal/mol), indicating that acetaminophen could spontaneously and strongly bind to the HK1 protein. Visualization with Discovery Studio 2019 revealed that acetaminophen stably occupied the active pocket of HK1 protein and formed hydrogen bonds with amino acid residues such as SER449, GLY233, THR232, and ASP209 to form stable hydrogen bonds (Fig. 5n), suggesting that these interactions constitute the primary binding forces. Furthermore, we utilized the Cistrome database to predict the transcription factors that potentially regulate *HK1* and performed molecular docking between acetaminophen and each factor. The results showed that acetaminophen was tightly bound to *EP300*, *ESR1*, *TRIM24*, *BRD4*, and *POLR2H*, with binding energies all less than −5 kcal/mol (Fig. 5o, p). Among them, the binding energies of *ESR1* and *EP300* were the lowest. Visual analysis showed that the ARG394 and LEU387 residuing on the *ESR1* receptor form hydrogen bond interactions with acetaminophen, and the GLU353, LEU349, LEU384, TRP383, ALA35, THR347, MET388, LEU346, PHE404, and LEU391 residuing on the *ESR1* receptor form acetaminophen van der Waals interaction force. The PRO1087 residued on the *EP300* receptor forms hydrogen bond interactions with acetaminophen, whereas ASP1088, ASP1091, TYR1089, GLU1113, GLN1112, GLN1116, GLN1082, PRO1081, and SER1046 residuing on the *EP300* receptor also form hydrogen bond interactions with acetaminophen van der Waals interaction force (Fig. 5q, r). In summary, acetaminophen not only directly binds HK1 to inhibit its transcription and expression but may also bind to *EP300* and *ESR1*, thereby epigenetically regulating the *HK1* expression.

### The *HK1*–*LDHA* axis mediates increased H3K18la modification in the *Wnt5a* promoter region of cartilage in male offspring rats induced by PAcE

**Fig. 6:**
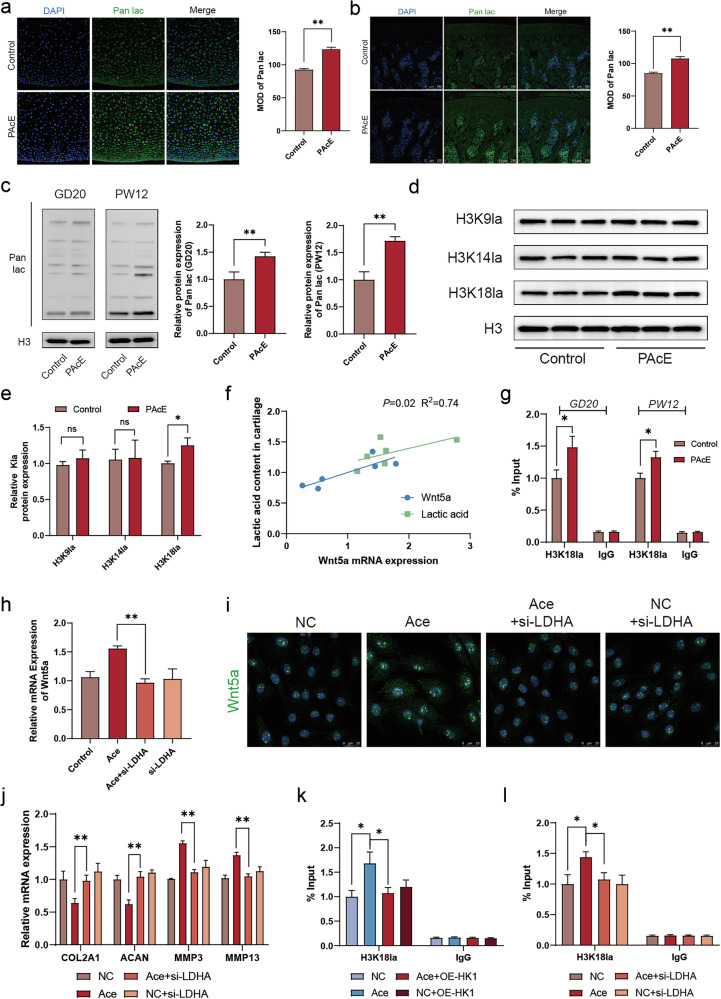
PAcE enhances glycolytic function and histone lactylation of *Wnt5a* in male offspring cartilages. **a**, **b** Representative immunofluorescence images of total histone lactylation in cartilage at GD20 and PW12 and their MOD quantification; scale bars, 75 μm and 250 μm, *n* = 5 biological replicates. **c** Western blotting (WB) images of the Pan Kla protein of cartilage at GD20 and PW12, and their statistical analysis; *n* = 3 biological replicates. **d** WB images of H3K9la, H3K14la, and H3K18la levels in fetal cartilage. **e** Statistical analysis of H3K9la, H3K14la, and H3K18la expression of above WB images, *n* = 3 biological replicates. **f** Correlation analysis of lactic acid content and *Wnt5a* mRNA expression in PAcE group. **g** ChIP-qPCR results of H3K18la expression in the *Wnt5a* promoter region of cartilage at GD20 and PW12, *n* = 6 biological replicates. **h**, **i** *Wnt5a* mRNA and protein expression after *LDHA* knockdown, scale bar, 25 μm, *n* = 6 biological replicates. **j** Effect of *LDHA* knockdown on mRNA expression of cartilage phenotype genes under the applying of acetaminophen, *n* = 6 biological replicates. **k**, **l** ChIP-qPCR results of H3K18la expression in the *Wnt5a* promoter region after *HK1* overexpression or *LDHA* knockdown, *n* = 6 biological replicates. All experiments were performed in technical triplicate. Values are expressed as means ± SEM. ^*^P < 0.05, ^**^P < 0.01 versus corresponding control. *ACAN*, aggrecan; Ace, acetaminophen; ChIP-qPCR, chromatin immunoprecipitation followed by PCR;*COL2A1*, collagen type II alpha 1 chain; DAPI, 4′,6-diamidino-2-phenylindole; GD20, gestational day 20; LDHA, lactate dehydrogenase A; LDHA-siRNA, silencing-lactate dehydrogenase A; MMP3, matrix metallopeptidase 3; MMP13, matrix metallopeptidase 13; MOD, mean optical density; NC, negative control; ns, not significant; PAcE, prenatal acetaminophen exposure; Pan lac, pan lactic acid-lysine; PW12, postnatal week; *Wnt5a*, wingless-type MMTV integration site family member 5a.

Thus, how does glycolysis influence *Wnt5a* expression? Recent studies have demonstrated that histone lactylation had a critical role in regulating gene expression and organ development^36^. We therefore hypothesized that acetaminophen-induced lactate accumulation in cartilage may alter gene expression through histone lactylation. We examined total lactylation levels in GD20 and PW12 cartilage. Pan-Kla levels were significantly elevated at both GD20 and PW12 (Fig. 6a–c). Furthermore, we next assessed three widely studied and readily modifiable histone lactylation sites — H3K18la, H3K9la and H3K14la. At GD20, H3K18la levels were significantly increased in the PAcE group, whereas H3K9la and H3K14la levels remained unchanged (Fig. 6d, e). Additionally, *Wnt5a* expression in PAcE cartilage was positively correlated with lactic acid content (Fig. 6f). We exposed the chondrocytes to lactate as an exogenous treatment. The CCK8 results showed that when the lactate concentration was lower than 8 mM, the viability of chondrocytes was not significantly affected (Supplementary Fig. 3a). In addition, 8 mM lactate treatment significantly upregulated the mRNA expression of *Wnt5a* (Supplementary Fig. 3b). The analysis of the Auto-Kla database (http://origin.tubic.org/Kla/public/index.php/index) revealed that the *Wnt5a* promoter region contains multiple lysine modification sites. Consistent with this, transcriptomic analysis showed significant differential expression of *Wnt5a* in the Wnt signaling pathway, and ChIP-qPCR confirmed that the enrichment of H3K9la and H3K14la in the *Wnt5a* promoter region showed no significant changes (Supplementary Fig. 3c, d), whereas the enrichment of H3K18la was significantly elevated at GD20 and PW12 in the PAcE group (Fig. 6g). Additionally, the treatment with exogenous lactate also increased the enrichment of H3K18la in the *Wnt5a* promoter region (Supplementary Fig. 3e). In summary, PAcE increases local lactic acid level in male offspring cartilage, thereby enhancing H3K18 lactylation in the *Wnt5a* promoter region.

We next transfected primary fetal chondrocytes with *LDHA*-siRNA to suppress glycolysis. Knockdown of *LDHA* reversed the acetaminophen-induced decrease in *COL2A1* and *ACAN* mRNA expression and the upregulation of *Wnt5a*, *MMP3*, and *MMP13* mRNA expression (Fig. 6h–j). In addition, in vitro ChIP-qPCR confirmed that acetaminophen increased H3K18la enrichment in the *Wnt5a* promoter (Fig. 6k, l), consistent with in vivo results. Moreover, both *HK1* overexpression and *LDHA* knockdown significantly reversed acetaminophen-induced increase in H3K18la enrichment in the *Wnt5a* promoter (Fig. 6k, l). The above indicates that the *HK1–LDHA* axis mediates the enhanced glycolytic feedback of primary fetal chondrocytes caused by acetaminophen, thereby increasing H3K18la enrichment in the *Wnt5a* promoter region.

## Discussion

Recent findings further support that OA has polygenic inheritance characteristics and intrauterine developmental origins^12,13^. Although epidemiological evidence links PAcE to various developmental risks, its long-term consequences on offspring cartilage remain unclear^37–39^. It is generally recognized that the duration of prenatal acetaminophen use does not typically exceed 3 days^40,41^, with an average daily dose of ~600 mg (maximum 2 g) in pregnant women^42^. Using the standard body surface area conversion formula for humans and rats (1:6.17), this dose corresponds to ~61.5–205 mg/kg per day in rats^43^. Our previous research indicated that PAcE during the second trimester causes fetal cartilage dysplasia in offspring mice, whereas PAcE during the third trimester has no significant effect on fetal cartilage development^22^. Therefore, in this study, we selected GD10-12 and administered 100 mg/kg per day acetaminophen by gavage for 3 consecutive days during mid-gestation to establish the PAcE rat model.

Furthermore, building on our team’s established two-hit hypothesis^13,14^, we found that mid-gestation PAcE not only compromises fetal cartilage matrix homeostasis but also exacerbates structural deterioration in adult male offspring following mechanical stress (long-distance running). This study elucidates a novel metabolism–epigenetics–gene axis: PAcE disrupts chondrocyte glycolysis via the *HK1*–*LDHA* axis, leading to lactate accumulation. This local metabolite mediates epigenetic reprogramming via specific H3K18 lactylation at the *Wnt5a* promoter, driving sustained *Wnt5a* activation and OA susceptibility with notable sexual dimorphism.

### *Wnt5a* upregulation mediates PAcE-induced chondrodysplasia and functional impairment

*Wnt5a* regulates physiological chondrocyte differentiation but promotes matrix metabolism impaired upon pathological activation^44^. Our findings identify *Wnt5a* as the critical effector linking intrauterine PAcE to adult cartilage dysfunction. Instead of a transient stress response, PAcE induced persistent *Wnt5a* upregulation prenatally and postnatally. Targeted intervention using AAV-sh*Wnt5a* effectively restored the matrix anabolic–catabolic balance in vivo, corroborating our in vitro observations. These findings position *Wnt5a* as a therapeutic target for intervention strategies. Notably, the chondrocyte-targeting AAV used in this study further enhanced the specificity of *Wnt5a* knockdown within the articular cartilage, and no obvious inflammatory response was detected following AAV delivery (Supplementary Fig. 4a–h). Moreover, although we cannot completely exclude the potential off-target effects associated with this technology, AAV-mediated intervention still exhibited a favorable effect in this study. We plan to further optimize the delivery system in future studies to minimize the limitations posed by off-target effects.

### The *HK1–LDHA* axis mediates the glycolytic metabolic reprogramming of chondrocytes induced by acetaminophen

Embryonic cartilage naturally develops in a hypoxic microenvironment, relying primarily on glycolysis for growth and differentiation^45,46^. Glycolysis is a metabolic pathway that converts glucose into pyruvate, generating ATP to support cellular energy demands while producing lactate as an end product^47^. Our in vitro experiments revealed that acetaminophen induced cartilage phenotype damage strictly under hypoxia, with no significant toxicity under normoxia, which explains the unique vulnerability of the cartilage developmental window. Mechanistically, acetaminophen suppresses *HK1* expression, which in turn triggers a specific compensatory hyperactivation of *LDHA* upregulation, lactate accumulation, and the elevated expression of *PFKM*, *PEPCK*, *PKM*, and glycoPER. Under hypoxic conditions, *HK1* overexpression successfully reversed the aforementioned results. Meanwhile, we found that *LDHA* upregulation is independent of the generic *HIF-1α* stress response, but rather a consequence of *HK1* suppression by acetaminophen. These results strongly indicated that acetaminophen may interact with *HK1*. Bioinformatics molecular docking predicted that acetaminophen directly binds to the *HK1* protein and its transcriptional regulators (*EP300*/*ESR1*). This prediction suggests that the drug suppresses *HK1* directly or indirectly at the transcriptional and translational levels.

These findings reveal a unique “compensatory hyperactivation” mechanism in PAcE-stressed chondrocytes, diverging from the classical pattern where *HK1* inhibition leads to global inhibition of glycolysis^1^. This “atypical” metabolic feedback pattern is an adaptive strategy for cells to maintain survival under energy stress and is the core linking PAcE, chondrocyte energy metabolism disorders, and subsequent epigenetic and gene expression disorders. In addition, this “metabolic reprogramming–disease progression” paradigm holds more broad developmental toxicological significance.

### H3K18 histone lactylation mediates persistent dysregulation of *Wnt5a* prenatally and postnatally

Epigenetic mechanisms have a significant role in cartilage development as well as in the onset and progression of OA^48^. Our previous studies demonstrated that gestational dexamethasone exposure induces offspring chondrodysplasia and adult OA susceptibility via *TGFβR1* promoter histone acetylation^49^ and is closely related to the phenotype stability of prenatal and postnatal cartilage. Recent research identifies lactate not merely as a cellular metabolic waste product but also as a direct substrate for histone lactylation to regulate gene transcription^50^, and histone lactylation has a critical role in embryonic development^51^.

In our in vivo model, fetal cartilage lactate content was elevated and strongly correlated with *Wnt5a* expression. We examined multiple lactylation marks to evaluate the site specificity. Although the total H3K18la level and its specific enrichment at the *Wnt5a* promoter increased in the PAcE offspring prenatally and postnatally, the levels of H3K9la and H3K14la at the same promoter remained unchanged. This contrast confirms that PAcE induced a highly specific H3K18la modification at the *Wnt5a* promoter. In vitro metabolic interventions provided the necessary orthogonal evidence. We administered exogenous lactate directly to the primary chondrocytes. Lactate supplementation directly increased the H3K18la enrichment at the *Wnt5a* promoter and upregulated its transcription. Furthermore, *LDHA* knockdown or *HK1* overexpression could significantly reduce the upregulation of H3K18la level in the *Wnt5a* promoter and cartilage damage caused by acetaminophen. It is suggested that the *HK1–LDHA* axis is crucial for controlling histone lactylation modifications in the *Wnt5a* promoter region of cartilage and for mediated persistent changes of cartilage quality of PAcE offspring prenatally and postnatally. It also underscores the mechanistic link between PAcE-induced epigenetic dysregulation and glycolytic reprogramming in cartilage. Collectively, these novel findings have translational relevance for prenatal medication safety assessment, therapeutic target identification for fetal-derived OA, and the development of early biomarker screening strategies.

### Sex-specific effects of PAcE on offspring cartilage development and adult OA susceptibility

Gestational adverse exposures frequently exert sex-specific effects on offspring disease susceptibility^52^. For instance, prenatal dexamethasone exposure preferentially impairs long bone development in male rather than in female fetal rats^53^. In this study, we found that PAcE-induced cartilage toxic in female offspring did not persist after birth. Epidemiological investigations and laboratory studies have shown that estrogen directly stimulates cartilage glycosaminoglycan synthesis in vitro^54^, and postmenopausal women show significantly higher OA incidence than age-matched men^55^, which suggests chondroprotective potential on its growth and development. In addition, placental factors contribute substantially to offspring sexual dimorphism^56^. Early placental developmental disturbances disproportionately affect female fetuses, whereas mid-late gestational insults preferentially impact males^57^. These findings may provide insights into explaining the sex differences observed in PAcE-induced chondrodysplasia and OA susceptibility. Nevertheless, the mechanisms underlying sex-specific vulnerability in fetal-origin diseases are highly complex and multifactorial, and this study did not further investigate these mechanisms. Addressing this question will be a major focus of future research.

This study provides the first evidence that PAcE impairs fetal cartilage development and increases OA susceptibility in offspring. Gestational acetaminophen suppressed chondrocyte *HK1* expression, triggering compensatory upregulation of downstream glycolytic genes and localized lactate overproduction. This metabolic shift enhanced H3K18la enrichment at the *Wnt5a* promoter, upregulating *Wnt5a* expression and ultimately disrupting chondrocyte matrix homeostasis, culminating in adult OA susceptibility. On the basis of these findings, we propose that *Wnt5a* could be a specific intervention target for the prevention and treatment of OA in female offspring of PAcE. Collectively, these findings inform the rational use of acetaminophen during pregnancy and provide theoretical and experimental basis for elucidating the etiology of fetal-derived OA and for developing early prevention strategies.

## Supplementary information

Supplementary Information

## Data Availability

The raw data supporting the conclusions of this article can be available from the corresponding author upon reasonable requests.
