# Supplementary material for: HK1–LDHA axis feedback regulation promotes histone lactylation of Wnt5a and mediates prenatal acetaminophen-induced susceptibility to osteoarthritis in male offspring
Source: Exp Mol Med. 2026 Jul 27;58(7):2392–406. doi: 10.1038/s12276-026-01793-1 (PMC13434236; doi:10.1038/s12276-026-01793-1)
Supplement: Supplementary file 1 [file 12276_2026_1793_MOESM1_ESM.pdf]

Supplementary Information

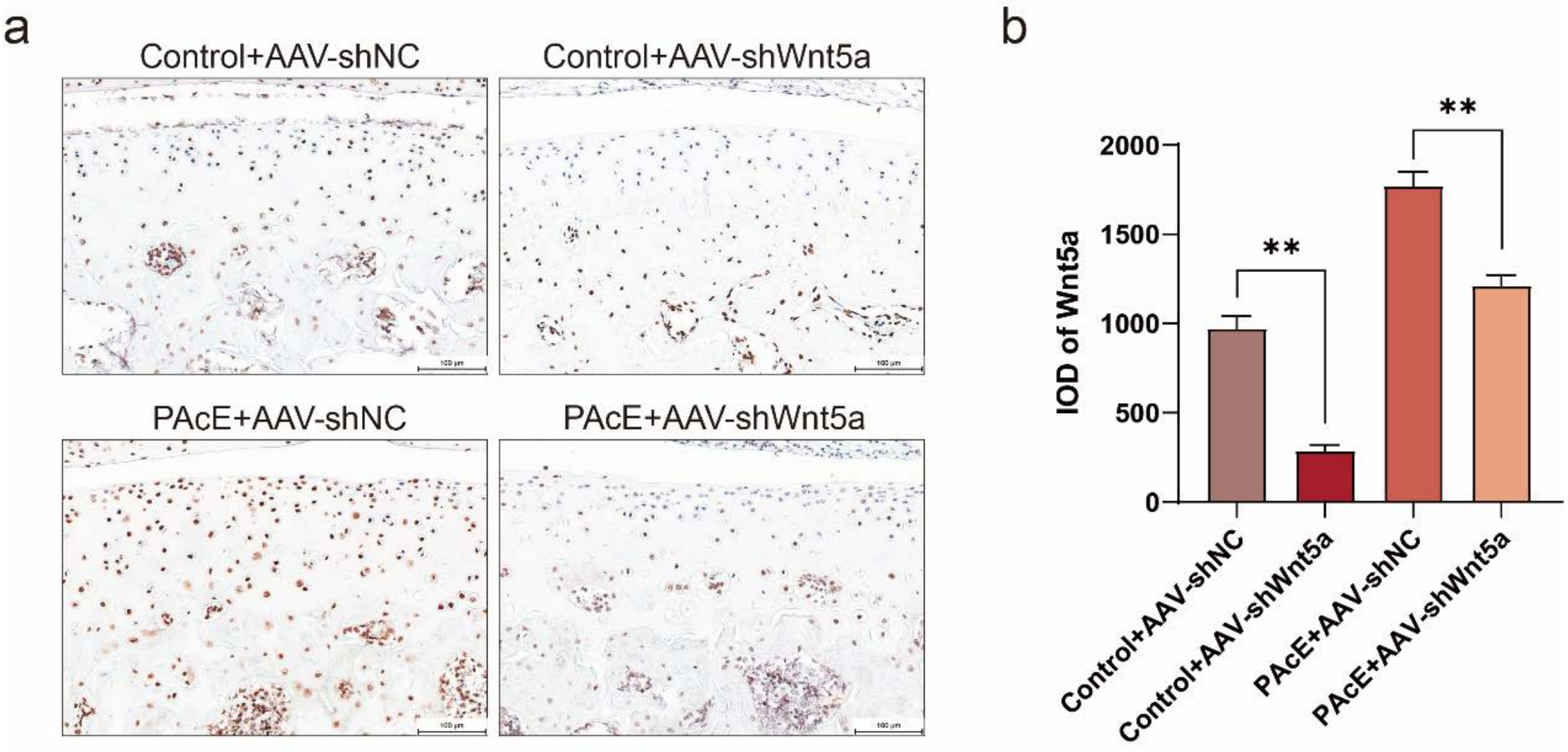

Supplementary Fig. 1. Injection of AAV-sh*Wnt5a* into the knee joint reduces the expression of Wnt5a in the cartilage of male offspring of PAcE. (a) Representative IHC images of Wnt5a in knee sections in Control+AAV-shNC, Control+AAV-sh*Wnt5a*, PAcE+AAV-shNC, and PAcE+AAV-sh*Wnt5a* groups; scale=100 μm; n=5 biological replicates; **(b)** Quantification of IOD values of the Wnt5a IHC; The experiment was performed in technical triplicate. Values are expressed as the means ± S.E.M. $^{*}P<0.05$, $^{**}P<0.01$ *vs*. corresponding control. PAcE, prenatal acetaminophen exposure; AAV, adeno-associated virus; shNC, short hairpin negative control; sh*Wnt5a*, short hairpin wingless- type mmtv integration site family member 5a; IOD, integrated optical density.

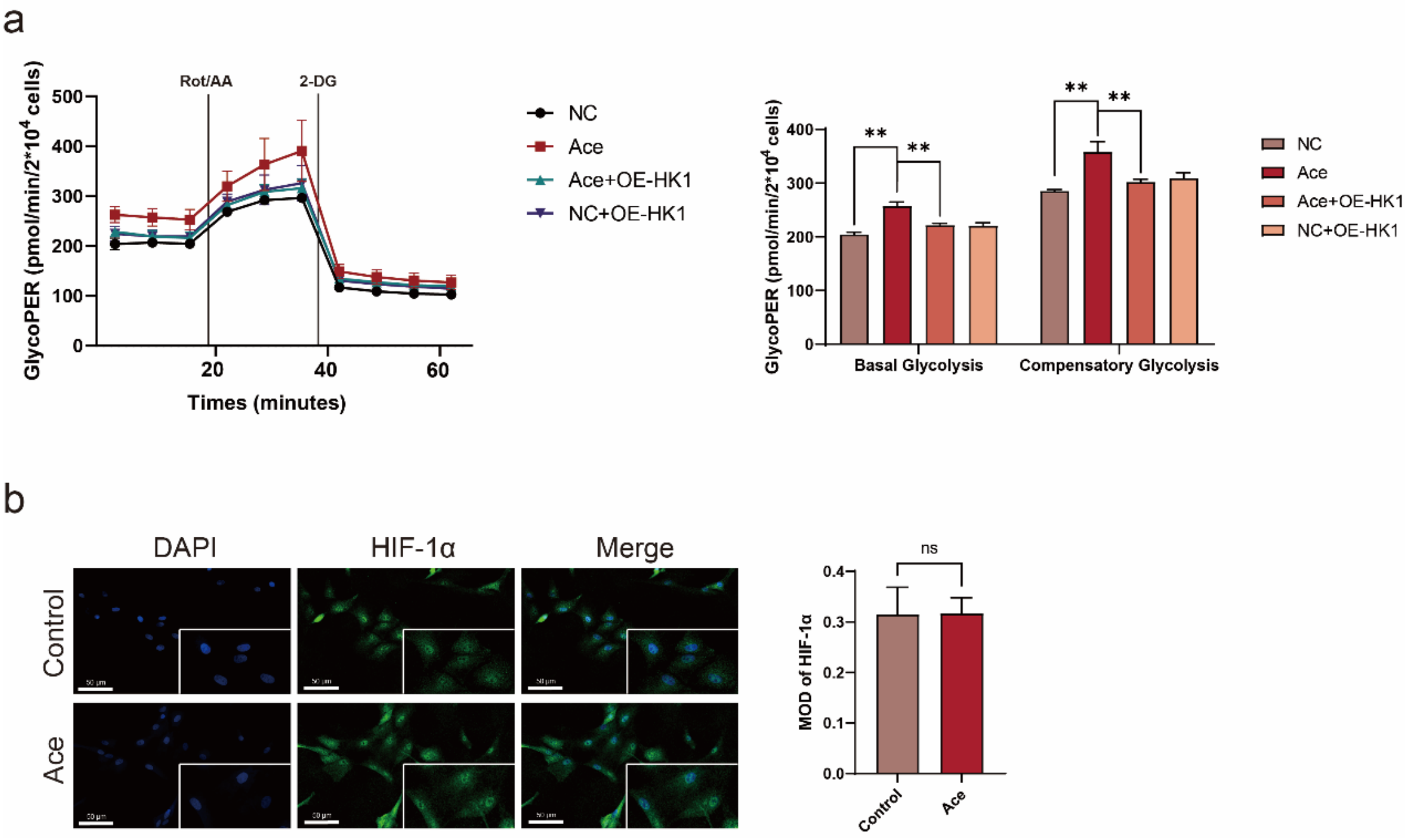

Supplementary Fig. 2. *HK1* mediates the increase in glycolysis rate of chondrocytes caused by Ace, as well as the effect of Ace on *HIF-1a*. (a) Real-time glycolytic proton efflux rate (glycoPER) treated as indicated in the figure, n=6 biological replicates; **(b)** Representative IF images of *HIF-1α and* quantification of MOD values, scale=50 μm, n=6 biological replicates; The experiment was performed in technical triplicate. Values are expressed as the means ± S.E.M. $^{*}P<0.05$, $^{**}P<0.01$ *vs*. corresponding control. *HK1*, hexokinase 1; *HIF-1α*, hypoxia inducible factor-1; Ace, acetaminophen; MOD, mean optical density.

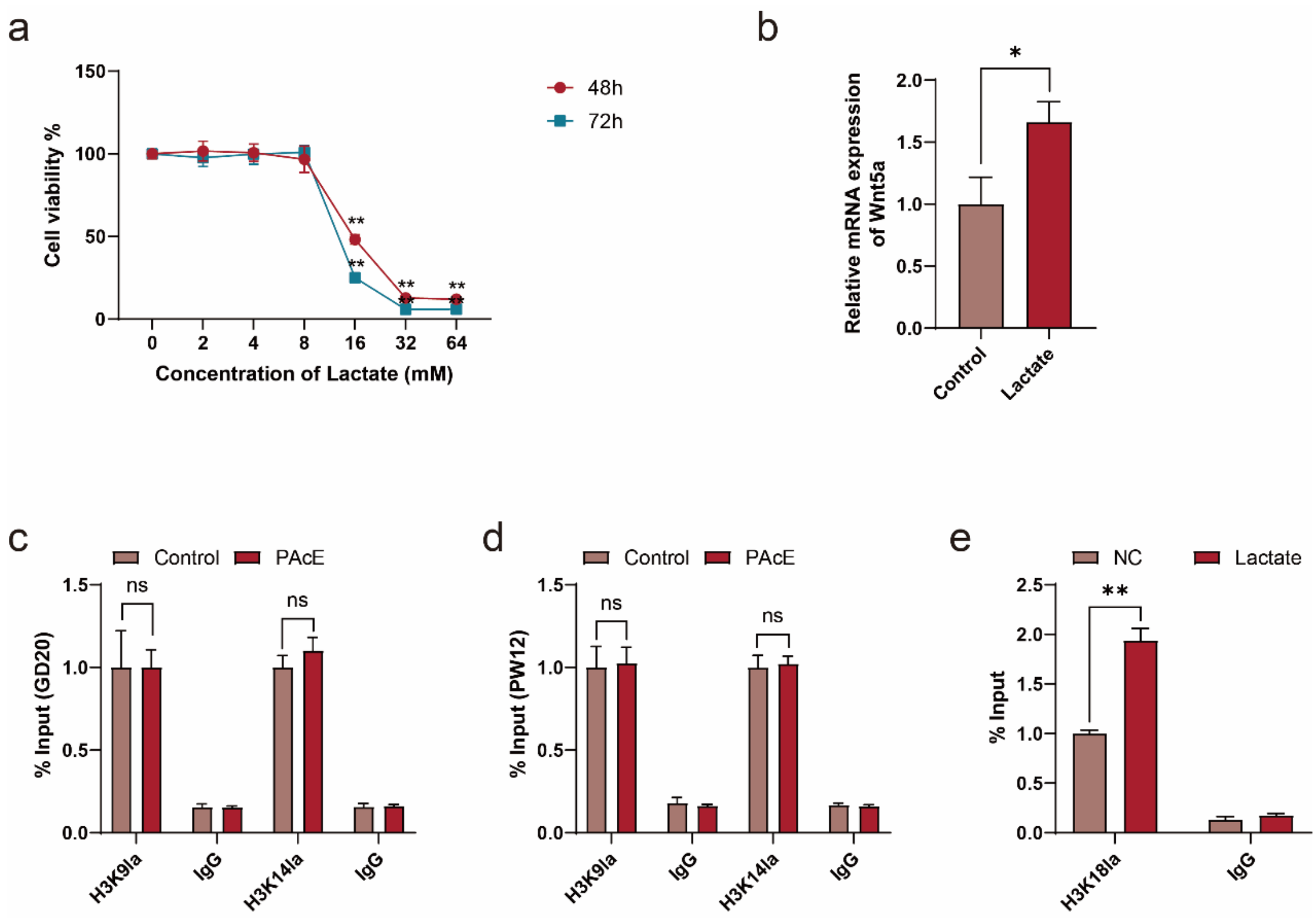

Supplementary Fig. 3. The changes in *Wnt5a* transcription levels and histone lactylation modifications in the lactate group and the PAcE group. (a) The CCK8 results of chondrocytes treated with lactate for 48 and 72 h under hypoxic conditions, n=6 biological replicates; **(b)** The mRNA expression of *Wnt5a* in chondrocytes under lactate treatment, n=6 biological replicates; **(c, d)** The enrichment of H3K9la and H3K14la in the *Wnt5a* promoter region of the PAcE offspring cartilage at GD20 and PW12, n=6 biological replicates; **(e)** The level of H3K18la in the *Wnt5a* promoter region of chondrocytes under lactate treatment, n=6 biological replicates; The experiment was performed in technical triplicate. Values are expressed as the means ± S.E.M. $^{*}P<0.05$, $^{**}P<0.01$ *vs*. corresponding control.

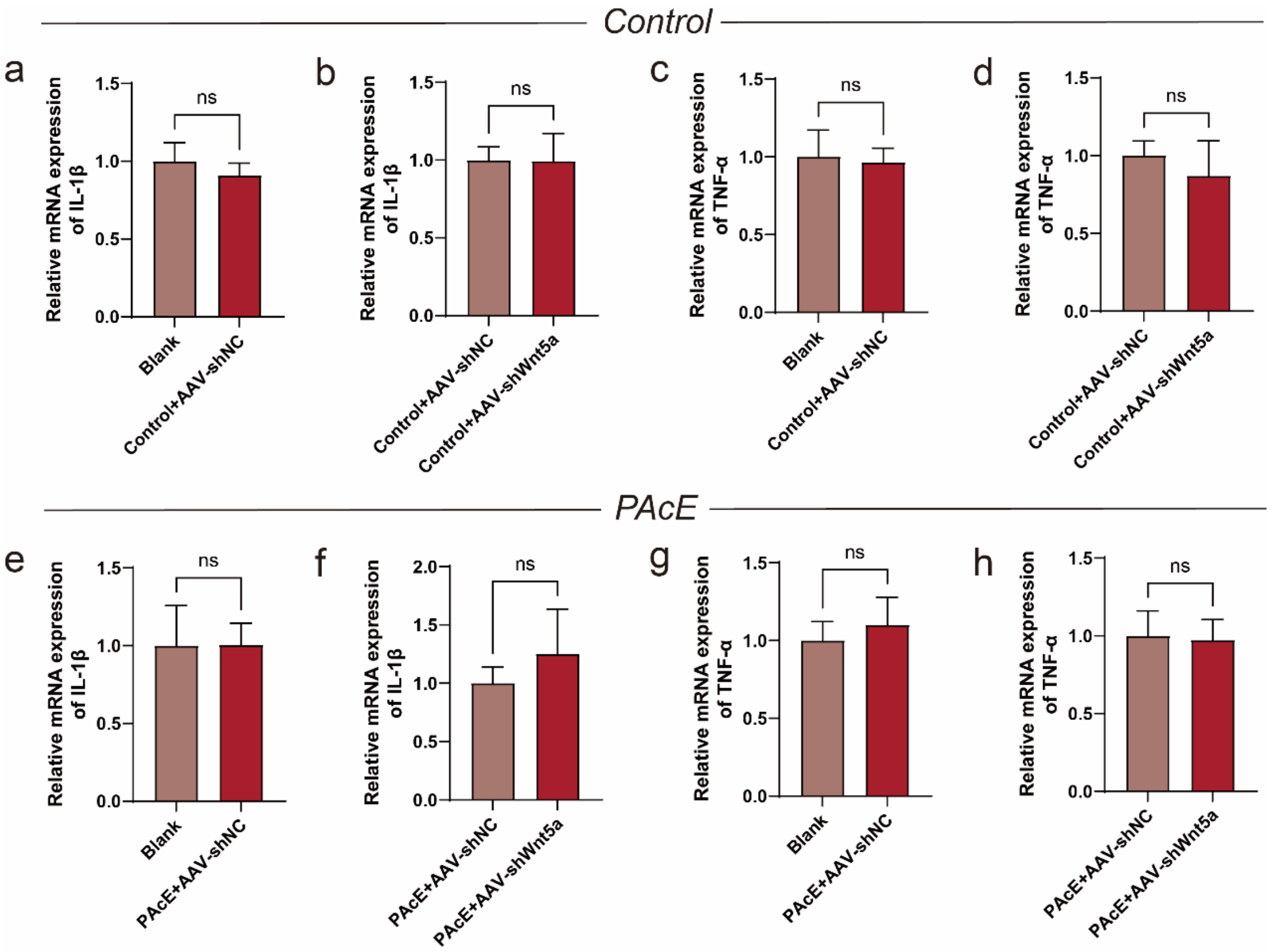

Supplementary Fig. 4. The effect of intra-articular injection of AAV virus on the expression of chondrocyte inflammatory factors. (a-d) The mRNA expressions of IL-1β and TNF-α in the Blank, Control+AAV-shNC and Control+AAV-shWnt5a groups, n=6 biological replicates; **(e-h)** The mRNA expressions of IL-1β and TNF-α in the Blank, PAcE+AAV-shNC and PAcE+AAV-shWnt5a groups, n=6 biological replicates; The experiment was performed in technical triplicate. Values are expressed as the means ± S.E.M. $^{*}P<0.05$, $^{**}P<0.01$ *vs*. corresponding control.
